# Mediastinal monophasic synovial sarcoma with vertebral metastases: A case report

**DOI:** 10.1002/ccr3.9303

**Published:** 2024-09-03

**Authors:** Miaomiao Men, Yousen Wu, Pengqi Tian, Changyou Long, Lei Zhou, Ting Fan

**Affiliations:** ^1^ Department of Radiology Affiliated Hospital of Qinghai University Xining China

**Keywords:** mediastinal, metastases, monophasic synovial sarcoma, rare, surgery

## Abstract

Mediastinal monophasic synovial sarcoma is a rare subtype that often lacks specific imaging characteristics, posing diagnostic challenges. This case report describes a mediastinal monophasic synovial sarcoma with vertebral metastasis, emphasizing imaging findings, differential diagnosis, and pathological features, thereby providing crucial support for accurate diagnosis and treatment planning.

## 1 INTRODUCTION

Synovial sarcoma (SS) accounts for 5%–10% of malignant soft tissue tumors.^1^ It is a mesenchymal spindle cell tumor with a certain degree of epithelial differentiation, which is classified as a soft tissue tumor of indeterminate differentiation, prone to recurrence and distant metastasis, and is a relatively rare malignant tumor. SS is characterized by clear boundaries, typically affecting individuals aged between 15 and 35 years, with the disease course lasting 2–4 years.^2^ In childhood and adolescence, SS is the second most prevalent malignant soft tissue tumors after rhabdomyosarcoma, and its pathogenesis may be related to trauma. Here, we report a case of mediastinal monophasic SS with vertebral metastases and describe its imaging manifestations on computed tomography (CT) and magnetic resonance imaging (MRI) examinations, pathological diagnosis, immunohistochemical findings, and postoperative follow‐up.

## 2 CASE HISTORY/EXAMINATION

The patient is a 14‐year‐old male who developed a chest wall tumor 5 years ago without any apparent cause, and the tumor has rapidly enlarged. He sought medical attention at the Thoracic Surgery Clinic of the Qinghai University Affiliated Hospital, where he was subsequently admitted for the treatment of a chest wall mass. Physical examination after admission revealed a rounded, elevated mass approximately 4 cm in diameter in the right scapular area. The tumor is soft to palpation, with limited mobility, and there is no tenderness on the sternum. The patient reports pain at the lesion site on the back, which is referred pain, predominantly occurring at night. Since the onset of the disease, the patient has been conscious and mentally capable, with no significant change in weight from before.

**FIGURE 1:**
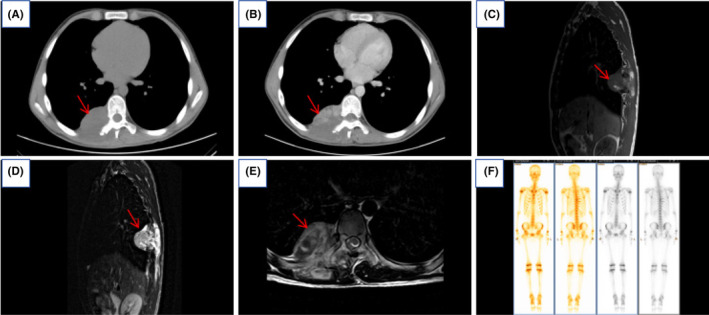
The enhanced scan of first computed tomography (CT) and magnetic resonance imaging (MRI) was admission: (A) The CT scan showed a right paravertebral mass at the level of the 8th and 9th thoracic vertebrae. (B) Enhanced CT scan showed marked inhomogeneous enhancement of the lesion, which is poorly demarcated from the right thoracic erector spinae muscle, with swelling and hypodensity of the right thoracic erector spinae muscle, and flocculent enhancement is seen within it (red arrows in A and B). MRI: (C) The T1WI sagittal lesion was isosignal with small patches of mixed high signal seen within it. (D) T2WI sagittal showed mixed signals with predominantly high signals. (E) T2WI cross‐sectional compression fat showed mixed signals of high and low with slightly high signals, and the lesion partially protruded into the spinal canal at the level of the thoracic 7 and 8 vertebrae, with compression of the spinal cord, and localized bone destruction of the right 8th and 9th posterior ribs and the right transverse process of the thoracic 8, with distended changes in the 9th posterior rib (red arrows in C, D and E). (F) Emission computed tomography (ECT) showed right 8 and 9 posterior ribs with localized concentration of bone nuclei and normal distribution of nuclei in the rest of the skeleton.

Chest CT revealed a mass‐like soft tissue lesion measuring approximately 6.06 cm × 5.69 cm at the level of the thoracic vertebrae 8 and 9, located in the right paraspinal region of the vertebral column. The lesion demonstrated marked inhomogeneous enhancement after contrast administration, with a CT value of about 71 HU. It was poorly demarcated from the adjacent right thoracic erector spinae muscle, which appeared swollen and hypointense. Striated and flocculent enhancement patterns were observed within it (Figure 1A,B). Thoracic MRI revealed a mass‐like lesion with mixed‐signal intensity, measuring approximately 5.98 cm × 5.62 cm, in the right paravertebral region at the T8 and T9 vertebral levels. In T1‐weighted images (T1WI), the lesion presented as isointense with small patches of mixed high signal within. In T2‐weighted images (T2WI), the lesion displayed mixed signals, predominantly high, along with areas of slightly higher signal intensity in T2WI fat suppression sequences. The lesion extended partially into the vertebral canal at the T7 and T8 levels, causing spinal cord compression. There was an evidence of partial destruction of the right 8th and 9th posterior ribs and the right transverse process of the 8th thoracic vertebra. Additionally, the 9th posterior rib appeared to be expanded (Figure 1C–E). Emission computed tomography (ECT) scan showed a clear skeletal image of the whole body with no structural abnormality. Bone scan findings showed increased uptake of radionuclides in the areas of the right 8th and 9th posterior ribs, while the distribution of radionuclides in the remainder of the skeletal system was normal (Figure 1F).

**FIGURE 2:**
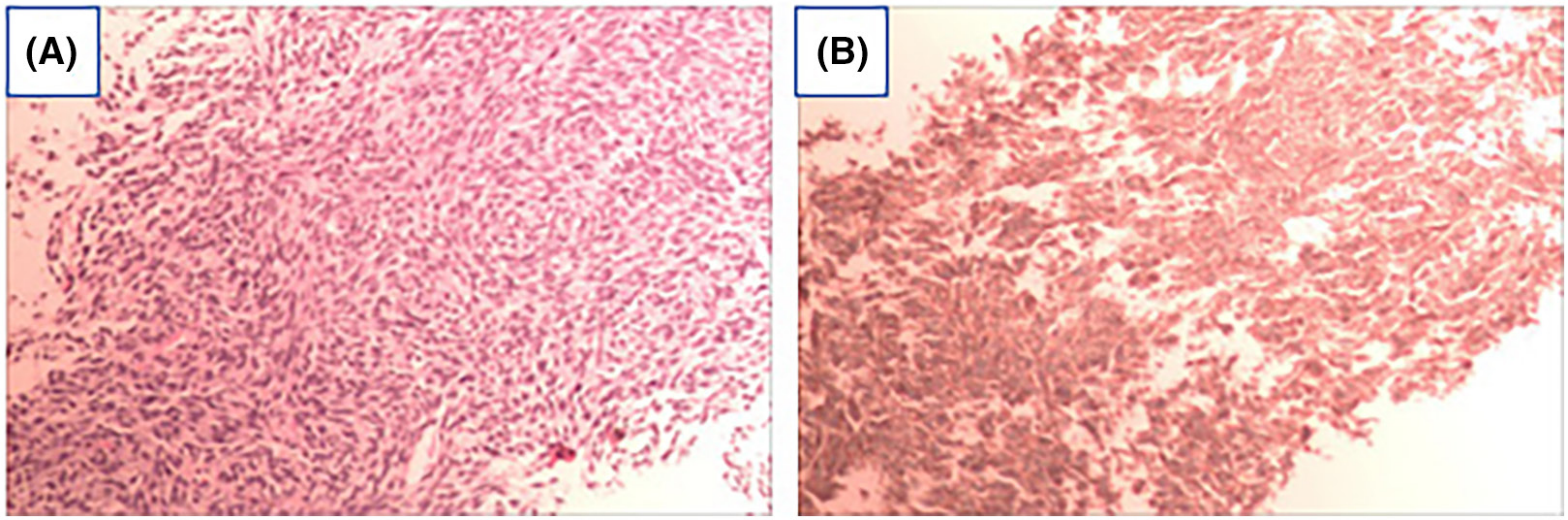
Ultrasound puncture biopsy of pathological tumor cells considering monophasic synovial sarcoma. (A) Microscopy (10 × 10) showed densely distributed short spindle cells with slit‐like structures and areas of the stroma exhibited myxoid changes. (B) tumor cells were stained by IHC and were focally positive for CD99.

The pathological analysis of the needle biopsy, performed under B‐ultrasound guidance, revealed densely packed tumor cells with a short spindle shape. Pathological mitotic figures and slit‐like structures were observed, and areas of the stroma exhibited myxoid changes. These findings are consistent with malignant soft tissue tumors, with monophasic SS being the primary consideration. Immunohistochemical results: AE1/AE3 (−), CD34 (vascular +), EMA (−), S100 (−), SMA (−), STAT6 (−), BcL‐2 (+), Ki67 (40%), CD99 (+), ERG (vascular endothelial +), CgA (−), SYN (−), NSE (−), Fli‐1 (−) (Figure 2).

## 3 METHODS (TREATMENT AND DIFFERENTIAL DIAGNOSIS)

**FIGURE 3:**
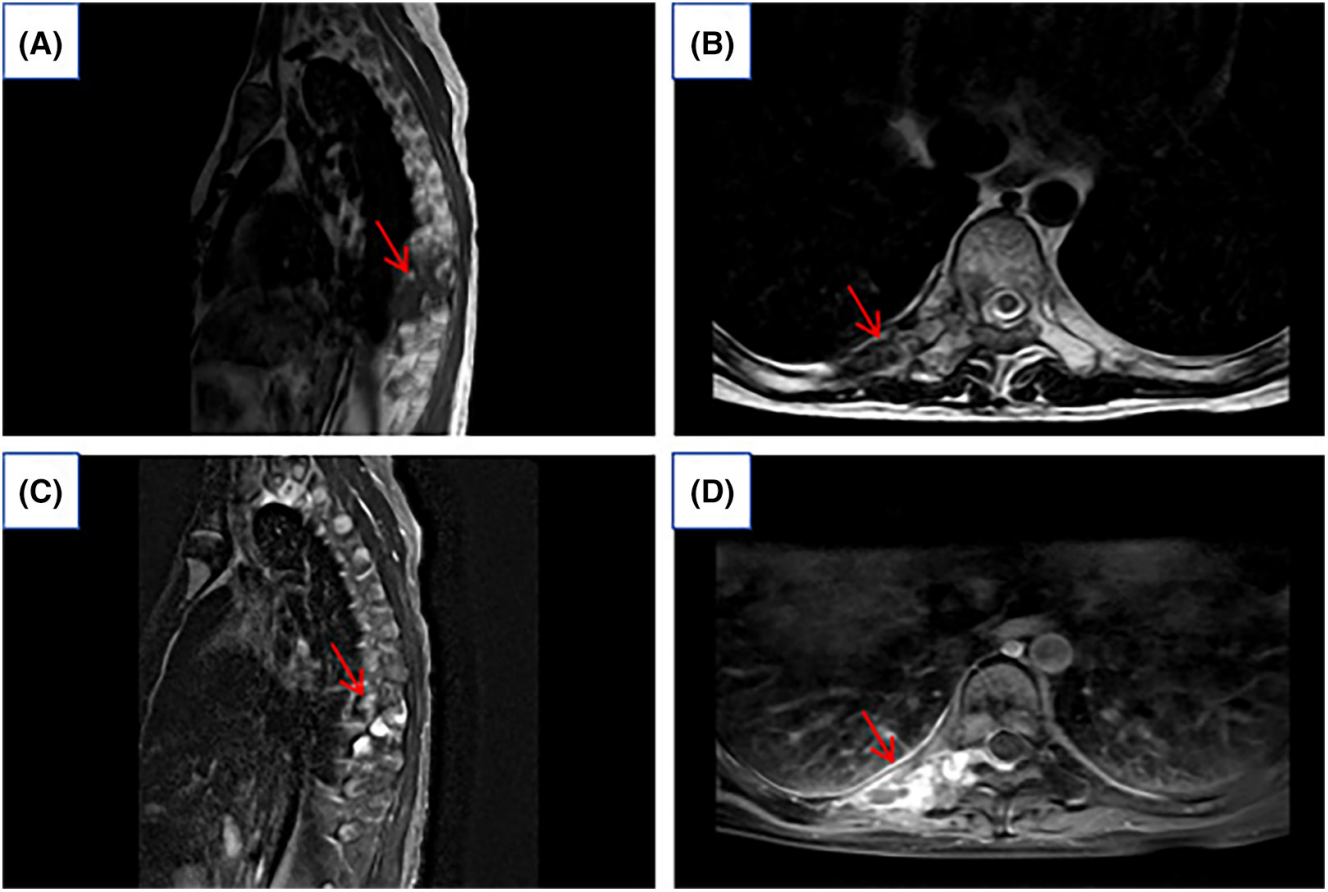
Preoperative MRI scanning with enhancement shows a mass‐like mixed signal shadow measuring approximately 2.42 cm × 1.37 cm. (A) The lesion was isosignal in the sagittal position of T1WI. (B) T2WI transects showed mixed high and low signals with predominantly high signals. (C) T2WI sagittal pressure lipids showed iso‐hypermixed signals with predominantly high signals. (D) T1WI transverse enhancement scan of the lesion showed marked inhomogeneous enhancement, within which streaks of unenhanced areas were observed (red arrows in A, B, C, and D).

The Department of Thoracic Surgery, considering the patient's young age and the extensive size of the tumor, noted concerns regarding potential trauma, intraoperative bleeding, and the possibility of residual tumor during surgery. Based on the pathological results, it was suggested to initiate targeted therapy with an anlotinib regimen, subsequently switching to pazopanib. After 2 years of radiotherapy, preoperative MRI scanning with enhancement shows the mass had reduced in size to 2.42 × 1.37 cm (Figure 3). There was an improvement in spinal cord compression and a reduction in the size of the intravertebral lesion. Consequently, the patient underwent surgery through a thoracic posterior approach for exploratory resection of a space‐occupying lesion on the thoracic wall and to repair the thoracic wall.

Monophasic SS needs to be differentiated from pigmented villous nodular synovitis (PVNS), fibrosarcoma, aggressive fibroma, and angiosarcoma according to imaging characteristics. PVNS typically presents as nodular or villous synovial thickening, with abundant hemosiderin deposition within the lesion. On MRI, it shows low to moderate signal intensity on both T1‐weighted and T2‐weighted images, and demonstrates marked enhancement post‐contrast.^3^ Fibrosarcoma presents as a soft tissue mass with minimal bone destruction and no significant calcifications visible on CT. Due to its fibrous composition, the lesion appears isointense to low signal on T1‐weighted images, while T2‐weighted images are more characteristic, showing a mixed high and low signal pattern reminiscent of brain gyri.^4^ These features can help distinguish it from monophasic SS.^5^ Invasive fibroma is the most common in middle‐aged people, and it occurs in the thigh, abdominal wall, and retroperitoneum. It usually has clear margins, lower density than muscle, and is more homogeneous. It has low signal in T1WI and T2WI due to rich fibrous components, and it shows progressive enhancement in enhancement scans.^6^ Enhanced MRI scans of monophasic SSs typically display uniform or slightly heterogeneous enhancement, unlike highly vascularized tumors such as angiosarcomas, which often exhibit significant heterogeneous enhancement on enhanced scans.^7^

## 4 CONCLUSION AND RESULTS (OUTCOME AND FOLLOW‐UP)

*Pathological diagnosis*: Mediastinal monophasic SS.

**FIGURE 4:**
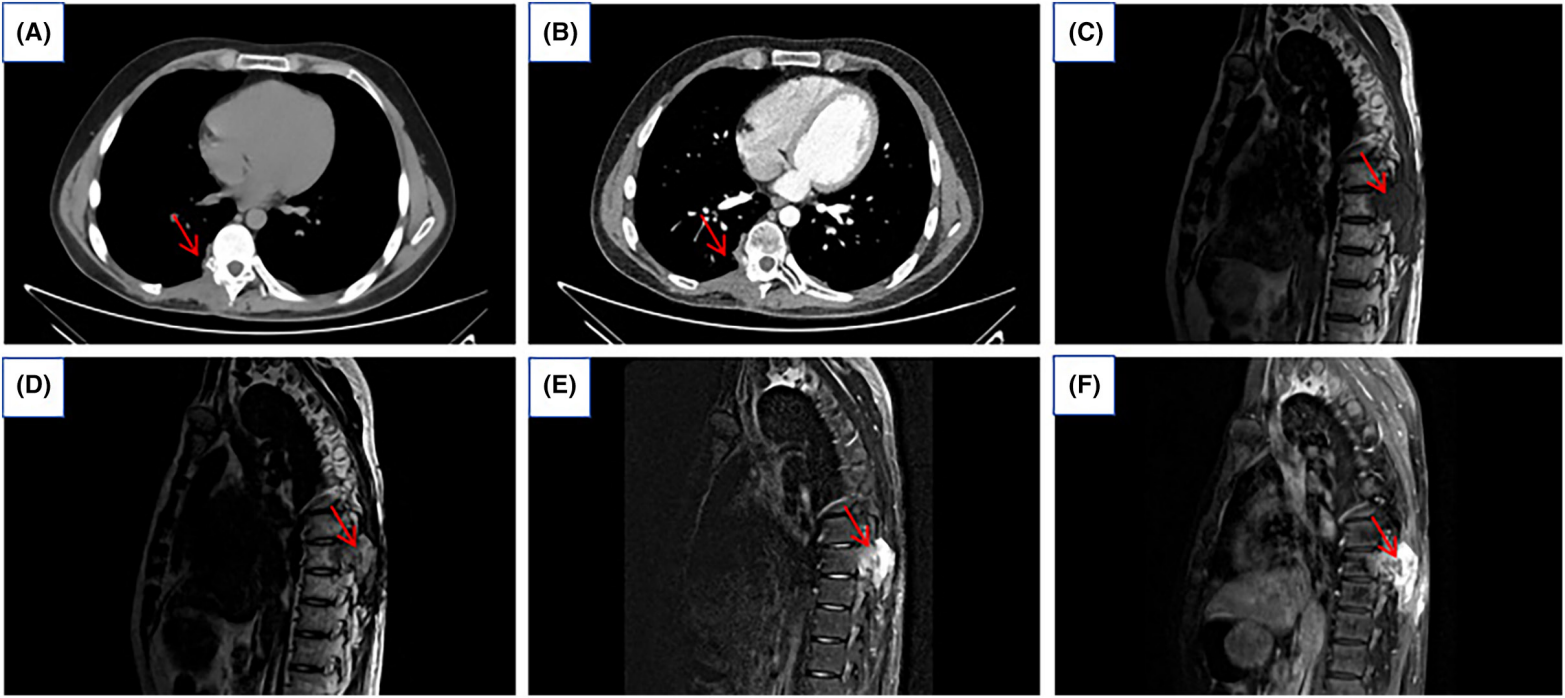
Six months postoperative follow‐up: (A) CT plain scan showed that some of the right‐sided arch boards of thoracic 8 and 9 and the right‐sided transverse processes, localized bony defects of the right‐sided 8th and 9th ribs show postoperative changes, and soft tissue thickening of the right‐sided trapezius and erector spinae muscles; (B) CT enhancement scan showed no abnormal enhancement (red arrows in A and B). Twelve months postoperative follow‐up: MRI showed a right paraspinal mass‐like mixed‐signal shadow at the level of thoracic 8 and 9 vertebrae; (C) T1WI sagittal lesion was isosinusoidal; (D) T2WI sagittal was isosinusoidal with a predominantly slightly higher signal; (E) T2WI sagittal compression lipids showed predominantly high signal with isohyperintense signal, and the lesion was poorly demarcated from the right thoracic erector spinae muscle. (F) T1WI sagittal enhancement scans of the lesion showed marked inhomogeneous enhancement, with small strips of unstrengthened areas within it, and the boundaries were poorly defined (red arrows in C, D, E, and F).

A follow‐up chest CT scan 6 months post‐surgery showed involvement of parts of the right arch board of thoracic vertebrae 8 and 9, and the right transverse process, with local bone defects in the right 8th and 9th ribs. The right trapezius and erector spinae muscles were thickened with soft tissue, but no other significant abnormalities were observed (Figure 4A,B). Twelve months post‐surgery, MRI indicated an increase in the extent of the lesion compared to previous images, showing signs of recurrence (Figure 4C–F).

## 5 DISCUSSION

SS is a type of soft tissue tumor soft tissue sarcoma (STS) with a high malignancy rate.^8^ It is a spindle cell tumor of mesenchymal tissue with some degree of epithelial differentiation, classified as an uncertainly differentiated soft tissue tumor. Morphologically, SSs can be histologically classified into three types: monophasic, biphasic, and poorly differentiated.^9^ SS was first reported in 1893 and occurs most frequently in the extremities^10^; Especially in the lower limbs below the knee, such as near the joint capsule and tendon sheaths of the foot, knee, or ankle joints.^11^ It can also occur in other parts of the body, including the trunk,^12^ head, and neck,^13^ abdomen,^14^ esophagus,^15^ and rectum.^16^ SSs occurring in non‐extremity sites have been reported only in isolated cases and have similar imaging appearances to SSs of the extremities. SS is distinguished from other soft tissue tumors by its low mean age of onset, which is common in young adults (mean age at diagnosis is 39 years) and is equally prevalent in both sexes.^1^ SSs have a tendency to be locally aggressive and metastatic, with 6%–18% of patients having metastases at the time of diagnosis. The majority of metastases occurred in the lungs (80%), followed by the bones (9.9%) and the liver (4.5%).^17^ This is a rare case of an adolescent male with a lesion located in the paramedian spine of the mediastinum with low morbidity and metastases to the vertebrae.

Imaging is necessary for the initial diagnosis, to understand the extent of tumor invasion, and assessing the effectiveness of treatment. Previous studies have shown that CT scans of monophasic SS usually depict a non‐uniform, non‐infiltrating mass with attenuation similar to or slightly below that of muscle, often surrounded by punctate calcifications. Calcification is common in lung metastases.^18^ Enhanced scanning helps to differentiate between cystic lesions or haematomas in SSs. SSs exhibit a variety of MRI manifestations, ranging from small, homogeneous nodules to large inhomogeneous masses encasing blood vessels and nerves.^5^ Monophasic SS typically appears as a heterogeneous, multifollicular soft tissue mass on MRI, and in up to 57% of cases there is a striking “triple sign,” that is, areas of mixed low, intermediate, and high signals, which result from a mixture of solid cellular components, hemorrhage or necrosis, and areas of calcification or fibrosis.^5^ Large cystic areas or hemorrhagic foci form a “grape bowl” sign, as evidenced by the lobulated margins of the lesion and the visible separation within the lesion.^19^ MRI enhancement scans show marked heterogeneous enhancement, with areas of necrosis, cysticity or hemorrhage that are not enhanced and areas of solidity that are enhanced.^20^ In this case, the CT scan of the lesion showed a soft tissue mass with mixed density and uneven enhancement on enhancement scan, with no obvious signs of calcification; the MRI T1WI lesion was isosignal, and small pieces of mixed high signal could be seen in the lesion; the T2WI showed mixed signals dominated by high signals; the T2WI compression lipids showed mixed signals dominated by slightly high signals, which were poorly delineated from the surrounding tissues, and manifesting as the “triple sign,” which was in line with the above study, and the case was accompanied by localized destruction of the bone quality of the adjacent vertebral body, expansive changes, and pathologic fracture, and the presence of metastatic signs.

In many cases, the diagnosis of SS requires not only the aid of imaging but also relies on morphology, immunohistochemistry (IHC) and molecular pathology.^21^ Histopathology remains the gold standard for monophasic SS. Monophasic SS is characterized by the presence of spindle cells showing moderate atypia, arranged in fascicles. Mast cells are a characteristic feature of SS.^22^ According to previous reports, monophasic SS typically shows positivity for vimentin (Vim), Bcl‐2, epithelial membrane antigen (EMA), TLE‐1, and CD99. Its spindle cells may also express keratin (CK) positivity. Additionally, some cases may exhibit focal positivity for S‐100.^23^ In contrast, fibrosarcoma typically shows negative expression of CK and EMA in IHC staining, with strong positive reactions for vimentin and CD34.^24^ However, a similar anti‐CD99 IHC membrane structure has been found in Ewing sarcoma.^23^ But monophasic SS is predominantly composed of spindle cells, typically exhibiting a spiral morphology and some degree of epithelioid differentiation, arranged in fascicles. In contrast, Ewing sarcoma often shows round cells arranged in nests or thin‐walled tubular structures, rather than a fascicular pattern, which aids in distinguishing between the two.^25^ The diagnostic findings of the pathology in this case, CD34 (vascular +), BcL‐2 (+), Ki67 (40%), CD99 (+), and ERG (vascular endothelial +), are generally consistent with the previous studies of monophasic SS.

In summary, mediastinal SS is a rare malignant mesenchymal tumor. Clinically, the main manifestation is an abnormally raised tumor mass, which may be accompanied by persistent or intermittent pain and swelling in the area of the tumor. If the tumor compresses nearby nerves, the patient may also experience tingling, numbness, or a decrease in function. Imaging examination is indispensable for detecting SS, providing the basis for surgery, and determining the prognosis. Histopathology is the most reliable method to diagnose this disease, and at present, the main treatment modalities are surgical resection and radiotherapy.

## AUTHOR CONTRIBUTIONS

**Miaomiao Men:** Conceptualization; data curation; formal analysis; investigation; methodology; resources; visualization; writing – original draft. **Yousen Wu:** Data curation; funding acquisition; resources; writing – review and editing. **Pengqi Tian:** Methodology; validation. **Changyou Long:** Formal analysis. **Lei Zhou:** Data curation. **Ting Fan:** Supervision.

## FUNDING INFORMATION

This research is supported by the Construction Project of National Clinical key Specialty.

## CONFLICT OF INTEREST STATEMENT

All authors have completed the ICMJE uniform disclosure form. The authors have no conflicts of interest to declare.

## ETHICS STATEMENT

The authors are accountable for all aspects of the work in ensuring that questions related to the accuracy or integrity of any part of the work are appropriately investigated and resolved. All procedures performed in this study were in accordance with the ethical standards of the institutional and/or national research committee(s) and with the Helsinki Declaration (as revised in 2013).

## CONSENT

This research was approved by the Medical Ethics Committee of the Affiliated Hospital of Qinghai University and exempted from subjects' informed consent, approval number: YJ‐SL‐2017. Written informed consent was obtained from the patient to publish this report in accordance with the journal's patient consent policy.

## Data Availability

All data for this study can be obtained by contacting the corresponding author.

## References

[1] Gazendam AM , Popovic S , Munir S , Parasu N , Wilson D , Ghert M . Synovial sarcoma: a clinical review. Curr Oncol. 2021;28:1909‐1920. 34069748 10.3390/curroncol28030177 PMC8161765

[2] Patrizi L , Borelli B , Di Prete M , et al. A rare case of vulvar superficial myofibroblastoma associated with ambigous and unusual differential diagnosis. Gynecol Oncol Rep. 2020;34:100637. 32953964 10.1016/j.gore.2020.100637 PMC7486683

[3] Nomura F , Ariizumi Y , Kiyokawa Y , et al. Pigmented villonodular synovitis occurring in the temporomandibular joint. Auris Nasus Larynx. 2019;46:609‐617. 30497770 10.1016/j.anl.2018.10.021

[4] Wang H , Nie P , Dong C , et al. CT and MRI findings of soft tissue adult Fibrosarcoma in extremities. Biomed Res Int. 2018;2018:6075705. 29693010 10.1155/2018/6075705 PMC5859867

[5] Fiore M , Sambri A , Spinnato P , et al. The biology of synovial sarcoma: state‐of‐the‐art and future perspectives. Curr Treat Options Oncol. 2021;22:109. 34687366 10.1007/s11864-021-00914-4 PMC8541977

[6] Subhawong TK , Feister K , Sweet K , et al. MRI volumetrics and image texture analysis in assessing systemic treatment response in extra‐abdominal desmoid fibromatosis. Radiol Imaging Cancer. 2021;3:e210016. 34213370 10.1148/rycan.2021210016 PMC8344342

[7] Gaballah AH , Jensen CT , Palmquist S , et al. Angiosarcoma: clinical and imaging features from head to toe. Br J Radiol. 2017;90:20170039. 28471264 10.1259/bjr.20170039 PMC5594986

[8] Dewi KP , Dewi IP , Iswanto I , Wulandari L . A review on pulmonary and mediastinal synovial sarcoma. J Basic Clin Physiol Pharmacol. 2023;34:169‐175. 36800987 10.1515/jbcpp-2022-0286

[9] Stacchiotti S , Van Tine BA . Synovial sarcoma: current concepts and future perspectives. J Clin Oncol. 2018;36:180‐187. 29220290 10.1200/JCO.2017.75.1941

[10] McCullough AE , Schwartz AJ , Taylor VL , Kransdorf MJ . Synovial sarcoma presenting as an avascular mass: radiologic‐pathologic correlation. Skeletal Radiol. 2015;44:279‐284. 25081635 10.1007/s00256-014-1964-9

[11] Smolle MA , Parry M , Jeys L , Abudu S , Grimer R . Synovial sarcoma: do children do better. Eur J Surg Oncol. 2019;45:254‐260. 30077520 10.1016/j.ejso.2018.07.006

[12] Fujibuchi T , Miyawaki J , Kidani T , et al. Intraosseous synovial sarcoma of the distal ulna: a case report and review of the literature. BMC Cancer. 2019;19:116. 30709383 10.1186/s12885-019-5325-x PMC6359868

[13] Sapalidis K , Laskou S , Manaki V , et al. Neck synovial sarcoma: case presentation. Rom J Morphol Embryol. 2021;62:575‐579. 35024747 10.47162/RJME.62.2.25 PMC8848270

[14] de Haas RJ , Bonenkamp JJ , Flucke UE , de Rooy JW . Synovial sarcoma of the abdominal wall: imaging findings and review of the literature. J Radiol Case Rep. 2015;9:24‐30. 10.3941/jrcr.v9i2.1992 PMC4391800 25926925

[15] Liddle S , Mitchell P . Synovial sarcoma of the oesophagus. ANZ J Surg. 2021;91:751‐753. 32857866 10.1111/ans.16237

[16] Williams PJ , Kwock C , Walker C , Walter O , Sucandy I , Chudzinski AP . Primary synovial sarcoma of the rectum. Am Surg. 2023;89:2893‐2896. 35147049 10.1177/00031348221074221

[17] Blay JY , von Mehren M , Jones RL , et al. Synovial sarcoma: characteristics, challenges, and evolving therapeutic strategies. ESMO Open. 2023;8:101618. 37625194 10.1016/j.esmoop.2023.101618 PMC10470271

[18] Bakri A , Shinagare AB , Krajewski KM , et al. Synovial sarcoma: imaging features of common and uncommon primary sites, metastatic patterns, and treatment response. AJR Am J Roentgenol. 2012;199:W208‐W215. 22826423 10.2214/AJR.11.8039

[19] Ashikyan O , Bradshaw SB , Dettori NJ , Hwang H , Chhabra A . Conventional and advanced MR imaging insights of synovial sarcoma. Clin Imaging. 2021;76:149‐155. 33607418 10.1016/j.clinimag.2021.02.010

[20] Liang C , Mao H , Tan J , et al. Synovial sarcoma: magnetic resonance and computed tomography imaging features and differential diagnostic considerations. Oncol Lett. 2015;9:661‐666. 25621034 10.3892/ol.2014.2774 PMC4301506

[21] Gronchi A , Miah AB , Dei Tos AP , et al. Soft tissue and visceral sarcomas: ESMO‐EURACAN‐GENTURIS clinical practice guidelines for diagnosis, treatment and follow‐up. Ann Oncol. 2021;32:1348‐1365. 34303806 10.1016/j.annonc.2021.07.006

[22] Thway K , Fisher C . Synovial sarcoma: defining features and diagnostic evolution. Ann Diagn Pathol. 2014;18:369‐380. 25438927 10.1016/j.anndiagpath.2014.09.002

[23] Mousavi SR , Farrokhi MR , Eghbal K , Dehghanian A , Rezvani A , Ghaffarpasand F . Metastatic thoracic and lumbar intramedullary and extramedullary Ewing's sarcoma: a rare case report and literature review. J Int Med Res. 2022;50. doi:10.1177/03000605221108095 PMC9364211 35938475

[24] Jiang H , Liu L , Li G . Primary synchronous ipsilateral renal fibrosarcoma and renal pelvic carcinoma: a case report and literature review. Onco Targets Ther. 2021;14:4119‐4125. 34262296 10.2147/OTT.S317094 PMC8275115

[25] Gajdzis P , Pierron G , Klijanienko J . Cytology of undifferentiated round‐cell sarcomas of bone and soft tissue: Ewing sarcoma or not Ewing sarcoma, that is the question. Acta Cytol. 2022;66:295‐306. 34515032 10.1159/000518146

